# Expression of inflammatory cytokines in CADASIL and their associations with clinical and neuroimaging features

**DOI:** 10.3389/fimmu.2025.1650847

**Published:** 2025-09-10

**Authors:** Li Bai, Haotian Yan, Zhongao Wang, Qing Peng, Haiqiang Jin, Yunchuang Sun, Fan Li, Wei Zhang, Zihao Zhang, Zhaoxia Wang, Yun Yuan, Hongjun Hao, Tao Wu, Chen Ling

**Affiliations:** ^1^ Department of Neurology, Peking University First Hospital, Beijing, China; ^2^ Beijing Key Laboratory of Neurovascular Disease Discovery, Beijing, China; ^3^ Rare Disease Medical Center, Peking University First Hospital, Beijing, China; ^4^ Beijing Anzhen Hospital Capital Medical University, Beijing, China; ^5^ State Key Laboratory of Brain and Cognitive Science, Institute of Biophysics, Chinese Academy of Sciences, Beijing, China; ^6^ University of Chinese Academy of Sciences, Beijing, China; ^7^ Anhui Province Key Laboratory of Biomedical Imaging and Intelligent Processing, Institute of Artificial Intelligence, Hefei Comprehensive National Science Center, Hefei, China

**Keywords:** CADASIL, inflammatory cytokines, TNF-β, MRI, CMBS

## Abstract

**Objectives:**

The aim of our study is to explore the expression levels of inflammatory cytokines in patients with cerebral autosomal dominant arteriopathy with subcortical infarcts and leukoencephalopathy (CADASIL), and to assess the correlation between inflammatory cytokines and clinical/magnetic resonance imaging (MRI) features of the patients.

**Methods:**

We recruited 54 patients with CADASIL and 28 healthy controls and detected the expression levels of the following inflammatory cytokines in peripheral blood: interferon (IFN)-γ, tumor necrosis factor (TNF)-α, TNF-β, interleukin (IL)-1β, IL-2, IL-4, IL-5, IL-6, IL-8, IL-10, IL-17F, and IL-22. We also analyzed the relationship between the expression levels of the inflammatory cytokines and the clinical/MRI features.

**Results:**

The expression of most inflammatory cytokines were significantly higher in CADASIL patients than in healthy controls, including IFN-γ (z = −5.335, *P* < 0.001), TNF-α (z = −4.880, *P* < 0.001), TNF-β (z = −2.401, *P* = 0.019), IL-1β (z = -2.831, *P* = 0.007), IL-4 (z = −4.039, *P* < 0.001), IL-5 (z = −4.523, *P* < 0.001), IL-6 (z = −3.545, *P* < 0.001), IL-8 (z = −5.667, *P* < 0.001), IL-17F (z = −3.986, *P* < 0.001) and IL-22 (z = −5.325, *P* < 0.001). The increased expression level of TNF-β was correlated with abnormal mRS scores in patients with CADASIL (odds ratio [OR] = 6.147, 95% CI: 1.324-28.535; *P* = 0.020) after adjustment for age, sex, history of hypertension and history of diabetes. The expression level of TNF-β was also associated with MMSE (β = -0.281, 95% CI:-5.325–0.866, *P* = 0.008) and apathy scores (β = 0.388, 95% CI:2.554-16.328, *P* = 0.008) after adjusting for age, sex, educational years, history of hypertension and history of diabetes. There was also a positive correlation between the expression level of TNF-β and the number of CMBs in deep (β = 0.314, 95% CI:2.989-39.461, *P* = 0.023) and lobar region (β = 0.433, 95% CI:15.363-59.857, *P* = 0.001) after adjusting for age, sex, history of hypertension and history of diabetes.

**Conclusions:**

Our results indicate that diffuse inflammatory pathway activation occurs in CADASIL. The increased expression level of TNF-β was associated with higher CMBs burden and poor clinical scores. Our findings suggest that the inflammatory pathway, particularly the TNF-related inflammatory pathway, may be involved in the disease progression of CADASIL.

## Introduction

Cerebral autosomal dominant arteriopathy with subcortical infarcts and leukoencephalopathy (CADASIL) is the most common hereditary cause of stroke (1), It is caused by mutations in the *NOTCH3* gene located on chromosome 19q12 (2). Magnetic resonance imaging (MRI) features of CADASIL include white matter hyperintensities (WMHs), lacune of presumed vascular origin (lacunes), and cerebral microbleeds (CMBs) (3). Clinically, CADASIL is characterized by migraine, recurrent stroke, cognitive impairment, and neuropsychiatric disorders (4).

Inflammatory cytokines play a significant role in the pathogenesis of cerebral small vessel disease (CSVD) (5, 6). Some studies have demonstrated that inflammatory cytokines are associated with the radiological progression of CSVD. For example, interleukin (IL)-6 has been linked to the formation of new lacunes in CSVD patients (7). Vascular cell adhesion molecule-1 (VCAM-1), a marker of vascular inflammation, has been found to be associated with WMHs in CSVD patients (8). The expression levels of vascular endothelial growth factor (VEGF) are also related to the formation of CMBs in CSVD patients (6). Additionally, certain inflammatory cytokines, such as tumor necrosis factor (TNF)-α, have been proposed to be associated with the clinical severity of CSVD, including cognitive decline (9). Fabry disease is a hereditary cerebral vascular disease caused by mutations in the *GLA* gene. Elevated expression levels of inflammatory cytokines, such as IL-1β or IL-6, are associated with the disease burden and clinical phenotype of Fabry disease (10–12).

In CADASIL, some studies have confirmed that inflammation may be involved in the progression of the disease. Ling et al. found that the activity of the NF-κB pathway is upregulated in vascular smooth muscle cells of CADASIL patients (13). Another study suggests the presence of systemic inflammation in CADASIL patients (14). However, the clinical significance of inflammatory cytokines in CADASIL remains unclear.

This study aims to analyze the expression levels of inflammatory cytokines in the peripheral blood of CADASIL patients and to reveal the relationship between these inflammatory cytokines and the patients’ clinical and imaging phenotypes.

## Methods

### Participants

This study was approved by the Institutional Review Board and Ethics Committee at Peking University First Hospital and was performed in accordance with the ethical standards laid down in the 1964 Declaration of Helsinki and its later amendments. Written informed consent was obtained from all subjects in this study. We recruited all patients with CADASIL who visited our department and met the following criteria, as well as healthy controls from the community, from August 2021 to November 2023. The inclusion criteria for patients with CADASIL were as follows: (1) confirmed genetic or pathological diagnosis of CADASIL, (2) no history of acute ischemic/hemorrhagic cerebrovascular events in recent three months, (3) >18 years old. Healthy controls had no known cerebrovascular disease (e.g. transient ischemic attack, stroke), no cardiac disease, no psychiatric illness, no major head trauma, or Alzheimer’s disease, as demonstrated by clinical interviews. Finally, a total of 54 patients and 28 age- and sex-matched healthy controls were included in our study.

The following clinical and demographic data were collected as in our previous study (15): age, sex, educational years, headache, migraine (16), transient ischemic attack (TIA)/stroke, history of hypertension, history of diabetes mellitus, and history of smoking/alcohol use were recorded. We also stratified patients based on the location of the variants (EGFr domains 1–6 vs. 7–34) (1). The degrees of dependence of all patients were determined by the modified Rankin scale (mRS). An mRS score ≥ 2 was defined as abnormal mRS (15). Mini-mental State Examination (MMSE) and Montreal cognitive assessment (MoCA) were used to evaluate overall cognitive efficiency. In addition, neuropsychiatric symptoms were assessed, including apathy and depression. Apathy was evaluated by the Apathy Scale (17). Depression was evaluated by self-rating depression scale (SDS) (18). Due to some patients being unable to complete the cognitive and neuropsychiatric scales, we ultimately collected MMSE data from 52 patients, MoCA data from 42 patients, and neuropsychiatric scores from 45 patients (**Supplementary Figure S1**).

### Peripheral blood inflammatory cytokines detection assay

Peripheral whole blood of all 54 patients and 28 healthy controls were collected in ethylenediaminetetraacetic acid tubes and immediately centrifuged at 1240 × g for 5 min at 4 °C. Plasma was isolated and frozen at −80 °C before further processing. Sample preparation and detection of cytokines—namely, interferon (IFN)-γ, TNF-α, TNF-β, IL-1β, IL-2, IL-4, IL-5, IL-6, IL-8, IL-10, IL-17F, IL-22—were performed using a sandwich enzyme-linked immunosorbent assay kit (914002, QuantoBio, Tianjin, China) following the manufacturer’s instructions. The cytokine concentrations of each sample were detected using a flow cytometer (BeamCyte-1026M, Jiangsu, China) and analyzed using CYTOSYS 2.0 software (Changzhou Bidako Biotechnology Co., Ltd., Changzhou, China).

### Quantification of MRI lesion burden

All patients underwent 7T brain MRI examination (7T whole-body MAGNETOM MR system, Siemens, Erlangen, Germany). The imaging parameters of all MRI sequences are summarized in **Supplementary Table S1**. Due to incomplete imaging data, two patients were excluded. Finally, we further analyzed the imaging data of 52 patients (27 males and 25 females). The MRI lesions of CADASIL patients, including WMHs, lacunes, and CMBs, were defined according to the STandards for ReportIng Vascular changes on nEuroimaging (19). The number of lacunes was counted manually. The severity of WMHs was assessed using the age-related white matter changes (ARWMC) score (20). The number of CMBs was calculated using the microbleed anatomical rating scale (MARS) (21). The ARWMC scores and the number of lacunes include basal ganglia and white matter regions, while the number of CMBs included both the deep (basal ganglia, thalamus, internal capsule, external capsule, corpus callosum and periventricular white matter) and lobar regions (frontal, parietal, temporal, occipital and insular lobes).

### Statistical analyses

Statistical analyses were performed with SPSS version 26.0 (SPSS, Inc, Chicago, IL). Box plots were used in this study to identify and remove outliers. The lower bound for outliers was defined as the first quartile minus 1.5 times the interquartile range (IQR), and the upper bound was defined as the third quartile plus 1.5 times the IQR. Any values falling below the lower bound or above the upper bound were considered outliers and were removed. The removed outliers were then imputed using multiple imputation techniques. Normally distributed data were compared using the independent samples t-test (t). Non-normally distributed data and small sample data were compared using the Mann-Whitney U test (z). The Chi-square (χ^2^) test was used to compare the ratios. The correlation between the mRS scores and inflammatory cytokines was first analyzed using univariate binary logistic regression. If the *P* value of the univariate binary logistic regression was < 0.05, multivariate binary logistic regression (forward LR) was further performed to adjust for age, sex, history of hypertension, and history of diabetes. The association between the MMSE/MoCA/apathy scores/SDS/ARWMC scores/number of lacunes/number of CMBs and inflammatory cytokines were first analyzed using univariate linear regression analysis. If the *P* value of the univariate linear regression was < 0.05, multivariate linear regression (stepwise) was further performed to adjust for age, sex, educational years, history of hypertension, and history of diabetes. The false discovery rate (FDR) adjusted *P*-value (*P*-adj) for multiple comparisons were used to estimate causal effects. Statistical significance was defined as *P* or *P-adj* < 0.05.

## Results

### Clinical manifestations and MRI features of patients with CADASIL

As shown in **Table 1**, a total of 54 patients with CADASIL (45.15 ± 10.39 years, 27 males and 27 females) and 28 healthy controls (41.25 ± 9.58 years, 15 males and 13 females) were included. There was no significant difference in age and sex between CADASIL patients and healthy controls. Among all CADASIL patients, headache, migraine, TIA/stroke, hypertension, diabetes mellitus was reported by twenty-two, twelve, thirty-nine, nine and three patients, respectively. Sixteen patients reported a history of smoking, and eleven reported a history of alcohol use. Abnormal mRS score was found in twenty-two patients. In terms of cognitive assessment, the average scores for the MMSE and the MoCA were 26.12 and 21.83, respectively. In addition, the average scores for neuropsychiatric symptoms, including apathy and depression, were 10.38 and 34.18, respectively. Among all CADASIL patients, the median value of ARWMC scores in the basal ganglia and white matter were 3.00 and 9.00, respectively. The median number of lacunes in the basal ganglia and white matter was 1.00 and 3.00, respectively. The median number of CMBs in the deep and lobar regions was 0.00 for both. **Table 1** presents the detailed clinical and neuropsychological characteristics of all patients.

**Table 1:** Demographic, clinical and MRI features of patients with CADASIL.

Variable	Patients with CADASIL
Age (mean ± SD, y)	45.15 ± 10.39
Sex (male/female)	27/27
Educational years (mean ± SD)	12.54 ± 4.25
History of headache, % (n)	40.74% (22)
History of migraine, % (n)	22.22% (12)
History of TIA or stroke, % (n)	72.22% (39)
History of hypertension, % (n)	16.67% (9)
History of diabetes mellitus, % (n)	5.56% (3)
History of smoking, % (n)	29.63% (16)
History of alcohol consumption, % (n)	20.37% (11)
mRS ≥ 2, % (n)	40.74% (22)
MMSE (mean ± SD)	26.12 ± 5.11
MoCA (mean ± SD)	21.83 ± 7.35
Apathy scale (mean ± SD)	10.38 ± 11.32
SDS (mean ± SD)	34.18 ± 9.18
ARWMC scores (median, range)	
Basal ganglia	3.00 (0-5)
White matter	9.00 (0-16)
Number of lacunes (median, range)	
Basal ganglia	1.00 (0-14)
White matter	3.00 (0-36)
Number of CMBs (median, range)	
Deep region	0.00 (0-151)
Lobar region	0.00 (0-269.5)

MRI, Magnetic resonance imaging; CADASIL, cerebral autosomal dominant arteriopathy with subcortical infarcts and leukoencephalopathy; TIA, transient ischemic attack; mRS, modified Rankin scale; MMSE, mini-mental state examination; MoCA, Montreal cognitive assessment; SDS, self–rating depression scale; ARWMC, age-related white matter changes; CMBs, Cerebral microbleeds.

### Peripheral blood inflammatory cytokine levels in patients with CADASIL

**Figure 1:**
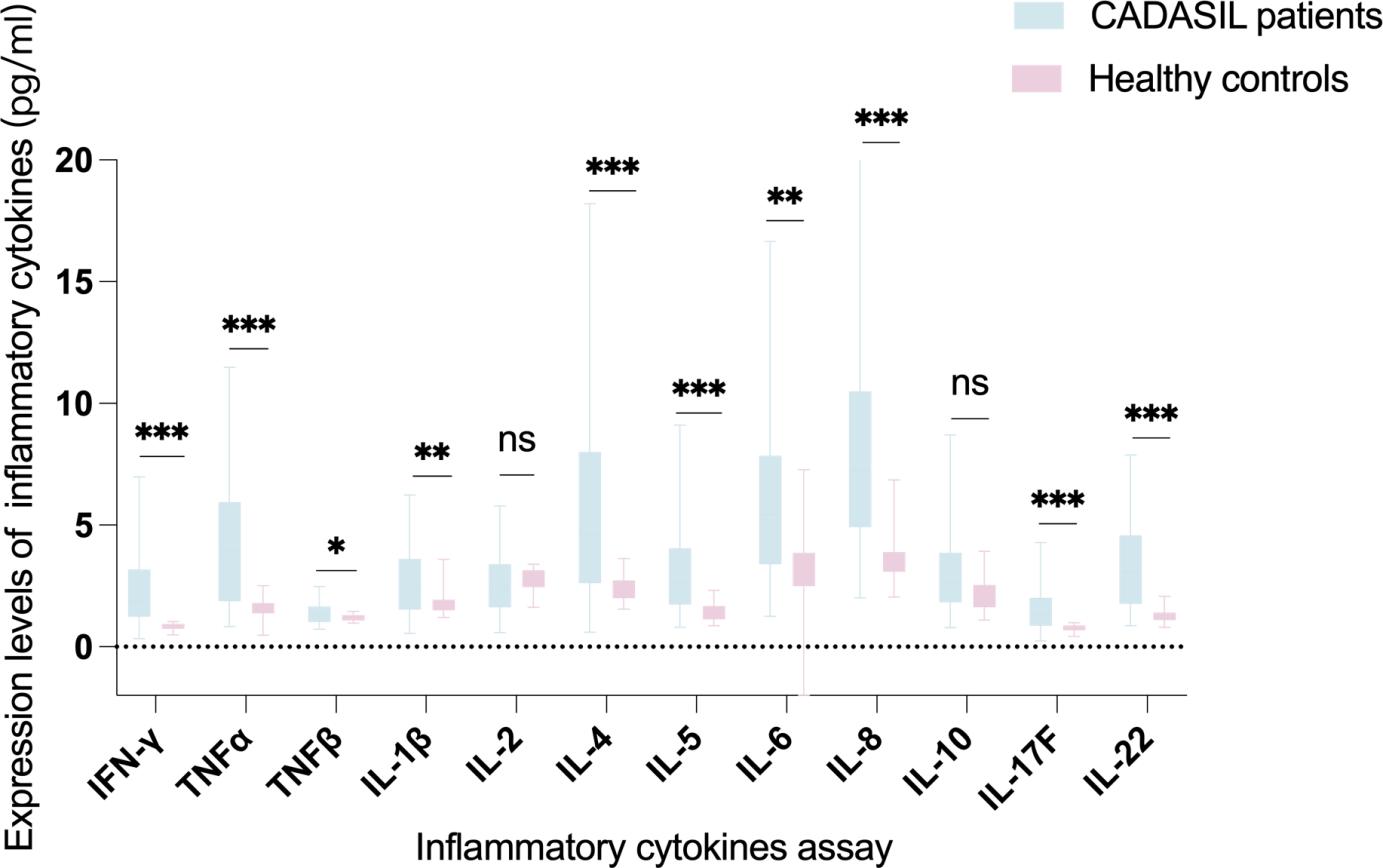
Comparison of peripheral blood expression levels of the twelve inflammatory cytokines between CADASIL patients and healthy controls. *P*-values were FDR-adjusted. Data was shown as median and range. ns, not significant. *P < 0.05, **P < 0.01, ***P < 0.001.

Most of the measured inflammatory cytokines showed elevated expression levels in CADASIL patients. After FDR correction, the levels of IFN-γ (z = −5.335, *P* < 0.001), TNF-α (z = −4.880, *P* < 0.001), TNF-β (z = −2.401, *P* = 0.019), IL-1β (z = -2.831, *P* = 0.007), IL-4 (z = −4.039, *P* < 0.001), IL-5 (z = −4.523, *P* < 0.001), IL-6 (z = −3.545, *P* < 0.001), IL-8 (z = −5.667, *P* < 0.001), IL-17F (z = −3.986, *P* < 0.001) and IL-22 (z = −5.325, *P* < 0.001) were significantly higher in the peripheral blood of CADASIL patients than in healthy controls. There were no significant differences in the levels of IL-2 (z = -0.944, *P* = 0.345) and IL 10 (z = -1.882, *P* = 0.065) between the two groups (**Table 2** and **Figure 1**).

**Table 2:** Comparison of peripheral blood expression levels of the inflammatory cytokines between CADASIL patients and healthy controls.

Inflammatory cytokines	CADASIL patients	Healthy controls	t/z/χ^2^	*P*	*P*-adj
Age	45.15 ± 10.39	41.25 ± 9.58	1.633	0.106	
Gender	27/27	15/13	0.094	0.759	
INF-γ	1.90 (0.34-6.98)	0.81 (0.49-1.04)	-5.335	<0.001^*^	<0.001^*^
TNF-α	3.92 (0.83-11.49)	1.53 (0.48-2.51)	-4.880	<0.001^*^	<0.001^*^
TNF-β	1.35 (0.72-2.48)	1.17 (0.98-1.46)	-2.401	0.016^*^	0.019^*^
IL-1β	2.50 (0.55-6.23)	1.71 (1.21-3.59)	-2.831	0.005*	0.007*
IL-2	2.39 (0.59-5.78)	2.72 (1.63-3.39)	-0.944	0.345	0.345
IL-4	4.58 (0.60-18.2)	2.33 (1.54-3.63)	-4.039	<0.001^*^	<0.001^*^
IL-5	2.61 (0.81-9.11)	1.41(0.87-2.33)	-4.523	<0.001^*^	<0.001^*^
IL-6	5.44 (1.25-16.65)	3.17 (-2.99-7.27)	-3.545	<0.001^*^	<0.001^*^
IL-8	7.25 (2.01-21.34)	3.44 (2.05-6.85)	-5.667	<0.001^*^	<0.001^*^
IL-10	2.63 (0.79-8.7)	2.07 (1.10-3.93)	-1.882	0.060	0.065
IL-17F	1.31 (0.25-4.28)	0.77 (0.43-0.99)	-3.986	<0.001^*^	<0.001^*^
IL-22	3.09 (0.87-7.88)	1.22 (0.81-2.08)	-5.325	<0.001^*^	<0.001^*^

INF, γ: interferon-γ; TNF, Tumor necrosis factor; IL, interleukin.

The expression of inflammatory cytokines was shown as median and range.

^*^Indicates a significant association. *P*-adj were FDR-adjusted.

We also stratified our cohort into two groups based on the location of the variants (EGFr domains 1–6 vs. 7–34). No statistically significant differences in peripheral inflammatory cytokine levels were observed between these groups (**Supplementary Table S2**).

### TNF-β expression level elevation is associated with increased CMBs burden and poor clinical scores

**Figure 2:**
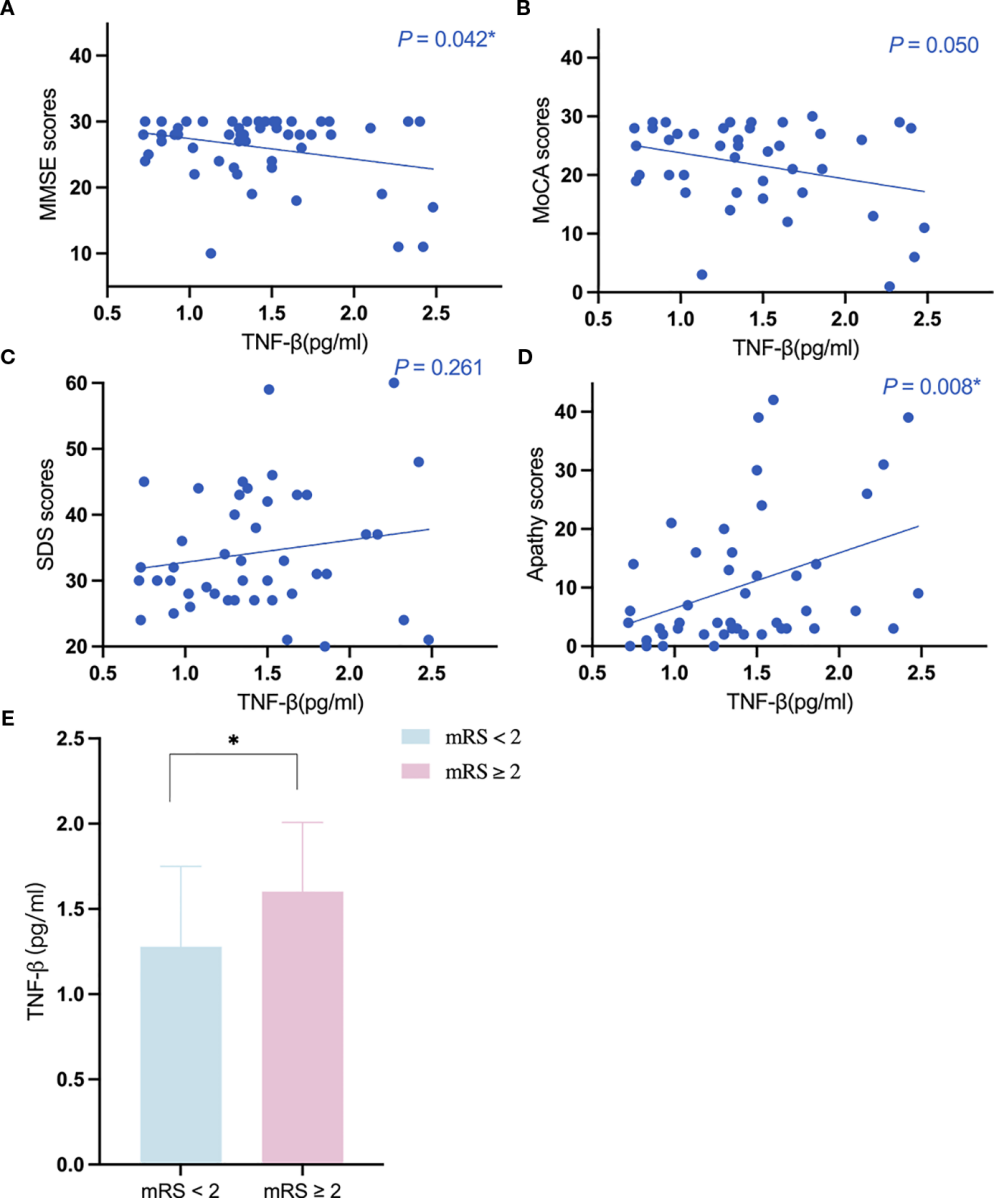
The relationship between the expression level of TNF-β and the key clinical markers. **(A, B)** The relationship between the expression level of TNF-β and cognition; **(C, D)** The relationship between the expression level of TNF-β and neuropsychiatric symptoms. **(E)** the expression level of TNF-β in different mRS subgroups. *Indicates P<0.05.

The results of the univariate regression analyses are detailed in the **Supplementary Table S3**-**S8**. The elevation of TNF-β expression level was associated with abnormal mRS scores in patients with CADASIL (odds ratio [OR] = 6.147, 95% CI: 1.324-28.535; *P* = 0.020), after adjustment for age, sex, history of hypertension, and history of diabetes. After adjustment for age, sex, educational years, history of hypertension, and history of diabetes, TNF-β expression level was significantly negatively correlated with MMSE scores (β = -0.281, 95% CI:-5.325–0.866, *P* = 0.008). TNF-β expression level was significantly positively correlated with apathy scores (β = 0.388, 95% CI:2.554-16.328, *P* = 0.008), after adjusting for age, sex, educational years, history of hypertension and history of diabetes. No correlation was found between the expression levels of other inflammatory cytokines and clinical scores (**Figure 2**).

**Figure 3:**
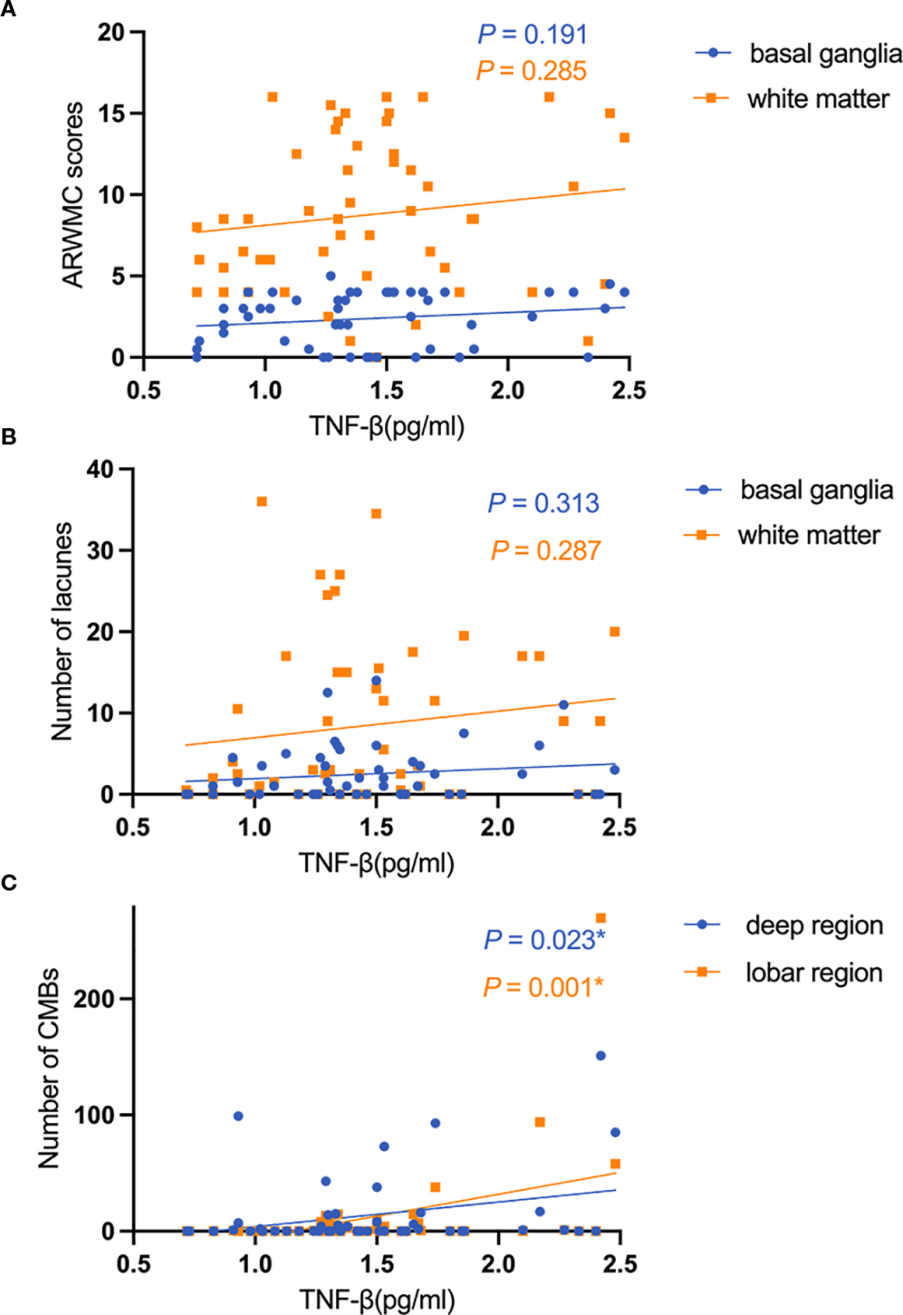
The relationship between the expression level of TNF-β and imaging markers. **(A)** The relationship between the expression level of TNF-β and ARWMC scores; **(B)** The relationship between the expression level of TNF-β and the number of lacunes; **(C)** The relationship between the expression level of TNF-β and the number of CMBs.

Additionally, after adjustment for age, sex, history of hypertension, and history of diabetes, TNF-β expression level was positively correlated with the number of CMBs in the deep (β = 0.314, 95% CI:2.989-39.461, *P* = 0.023) and lobar regions (β = 0.433, 95% CI:15.363-59.857, *P* = 0.001). However, the expression level of TNF-β was not associated with the ARWMC scores and the number of lacunes in CADASIL patients. In contrast, the expression level of IL-6 was only associated with the number of CMBs in the deep region (β = 0.295, 95% CI:0.200-4.712, *P* = 0.033). No correlation was found between other cytokines and MRI burden (**Figure 3**).

## Discussion

Our results indicate that diffuse inflammatory pathway activation occurs in CADASIL, involving pro-inflammatory cytokines such as IL-1β, IL-5, IL-6, IL-8, L-17F, IL-22, TNF-α, TNF-β, and IFN-γ (produced by T cells and monocytes/macrophages) and anti-inflammatory cytokines such as IL-4 (produced by monocytes/macrophages). Among them, the increased expression level of TNF-β was associated with higher CMBs burden and poor clinical scores. Our findings suggest that the inflammatory pathway, particularly the TNF-related inflammatory pathway, may be involved in the disease progression of CADASIL. The TNF-related inflammatory pathway may be a promising therapeutic target for CADASIL.

In this study, compared with healthy controls, the expression levels of most inflammatory cytokines were significantly increased in CADASIL patients. Previous studies have detected systemic inflammatory cytokines, including IL-1β, IL-6, TNF-α, CCL2, and CXCL16, and found that their expression levels were significantly elevated in CADASIL patients, which is consistent with our results (14). The elevation of inflammatory cytokines can also be observed in CSVD. Wu et al. found that the expression levels of serum IL-6, TNF-α, and C-reactive protein (CRP) were significantly elevated in patients with CSVD (9). Additionally, in CSVD, the neutrophil-to-lymphocyte ratio (NLR), a systemic inflammatory marker, was also elevated (22). Elevated levels of IL-6 and TGF-β1 were also found in patients with Fabry disease, suggesting that chronic inflammation is a driver of organ damage in Fabry disease (23). Meanwhile, vascular inflammation plays an important role in the progression of CSVD and Fabry disease. In patients with CSVD, vascular inflammation may lead to damage to brain regions supplied by perforating arteries (6), and the mechanisms involved may include oxidative stress, vascular endothelial dysfunction, and narrowing of the lumen, ultimately leading to the progression of CSVD (24). In Fabry disease, elevated levels of inflammatory cytokines are also associated with disease severity (25). A study based on CADASIL-induced pluripotent stem cells has shown that there is activation of the NF-kB pathway in CADASIL vascular smooth muscle cells, and CADASIL endothelial cells are more likely to recruit inflammatory cells under the action of pro-inflammatory cytokines (13). These studies further corroborate the possible activation of inflammatory pathways in CADASIL and may be associated with the occurrence and development of the disease, providing a theoretical basis for our results.

We found that in CADASIL patients, the expression level of TNF-β was associated with the burden of CMBs and clinical scores, suggesting that TNF-β may be a potential candidate biomarker reflecting disease burden and severity in CADASIL. Similarly, in CSVD, other studies have further confirmed the correlation between inflammatory cytokines and MRI burden. One study in CSVD found that increased expression of systemic inflammatory biomarkers, including CRP, IL-6, and TNF-α, was associated with a reduced number of deep medullary veins (26). Another study revealed that elevated levels of high-sensitivity IL-6 in peripheral blood, as well as increased expression of IL-8 and IL-17 in monocytes, were associated with the progression of WMH volume in CSVD patients (27). In addition, increased expression of other inflammatory markers, such as intercellular adhesion molecule 1, and decreased levels of myeloperoxidase, were associated with more severe WMH and the occurrence of cerebral infarctions in CSVD patients (28). In CSVD, inflammatory cytokines are also correlated with clinical scores. Elevated levels of neutrophil extracellular traps (NETs) were significantly associated with worse MoCA scores among CSVD patients, indicating that NETs may be a potential biomarker for CSVD-related cognitive impairment (29). Another study measured serum levels of soluble TNF receptor 1 in CSVD patients and also found that higher levels of soluble TNF receptor 1 were associated with cognitive decline (30).

TNF-β, also known as lymphotoxin-α (LT-α), is a member of the tumor necrosis factor superfamily (TNFSF) and is a cytokine produced by lymphocytes (31). TNF-β plays an important role in various biological processes such as immune regulation, inflammatory responses, and apoptosis. The TNF-β related signaling pathways are closely related to the occurrence and development of many diseases, such as autoimmune diseases (rheumatoid arthritis, Crohn’s disease) (32), neurological diseases (multiple sclerosis, Alzheimer’s disease, Parkinson’s disease) (33, 34), cardiovascular diseases (atherosclerosis, aortic dissection) (35), and cancer (36). The NF-κB signaling pathway plays a crucial role in the inflammatory response. Upon stimulation, activated NF-κB translocates to the nucleus, promoting the expression of genes encoding cytokines (37). Previous studies have demonstrated that NF-κB is a key regulatory factor controlling the inducible expression of the TNF-β gene (38). The NOTCH signaling pathway is a highly conserved intercellular communication mechanism in multicellular organisms, playing a key role in the development of organisms and the determination of cell fate. The NOTCH signaling pathway interacts extensively with other signaling pathways such as Wnt, TGF-β, and NF-κB (39–41). The vascular lesions in CADASIL are caused by mutations in the NOTCH3 gene, and previous studies have confirmed the overactivation of the NOTCH pathway in CADASIL (42). Previous research has also confirmed the activation of NF-κB in the vascular smooth muscle cells of CADASIL, and this activation may be mediated by NOTCH pathway (13). Another study also identified the importance of NOTCH3 for the activation of NF-κB and pro-inflammatory development (41).Therefore, the NOTCH signaling pathway may promote the expression of inflammatory factors such as TNF-β through its interaction with the NF-κB pathway.

### Limitations

However, the study has several limitations. First, because our study focused on a rare disease, the sample size was inevitably small, limiting the generalizability of our findings. These results should therefore be validated in larger, multicenter cohorts. Second, because we only included twelve inflammatory cytokines, the entire immune process may not be captured. Third, due to the lack of longitudinal study data, a causal relationship between inflammatory factors and disease severity cannot be clearly established. Lastly, our study involved the expression of multiple inflammatory cytokines, which may interact in a complex network, and further research is needed to explore the significance of inflammatory pathways in CADASIL disease.

## Conclusions

In conclusion, our study demonstrated that the expression levels of peripheral blood inflammatory cytokines were widely upregulated in CADASIL patients compared with healthy controls. Moreover, the elevated expression level of TNF-β was associated with a higher burden of CMBs and poor clinical scores. Overall, our findings suggest that inflammatory pathways may be involved in the progression of CADASIL disease, and the expression level of peripheral blood TNF-β may serve as a biomarker for assessing the condition of patients with CADASIL.

## Data Availability

The original contributions presented in the study are included in the article/**Supplementary Material**. Further inquiries can be directed to the corresponding authors.
