# Supplementary material for: Expression of inflammatory cytokines in CADASIL and their associations with clinical and neuroimaging features
Source: Front Immunol. 2025 Sep 10;16:1650847. doi: 10.3389/fimmu.2025.1650847 (PMC12457143; doi:10.3389/fimmu.2025.1650847)
Supplement: Supplementary file 1 [file DataSheet1.docx]

**Supplementary material**

**
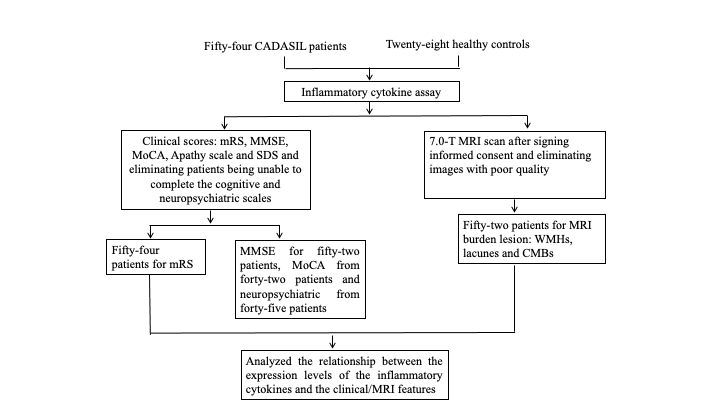
**

Figure SI

The flow diagram of the study

**Table SI. The imaging protocols of MRI sequences.**

| Sequence | Resolution (mm^3^) | TR (ms) | TE (ms) | FA (deg) |
| --- | --- | --- | --- | --- |
| T1w-MPRAGE | 0.7×0.7×0.7 | 3000 | 3.76 | 8 |
| FLAIR | 0.8×0.8×0.8 | 8000 | 400 | 120 |
| T2*w-GRE | 0.40×0.40×0.80 | 36 | 5.98, 12.14, 18.3, 24.46, 30.62 | 14 |

Abbreviations: MRI: Magnetic resonance imaging, MPRAGE: magnetization-prepared rapid gradient echo; FLAIR: fluid-attenuated inversion recovery; GRE: gradient echo; TR: repetition time; TE: echo time; FA: flip angle.

**Table SII. Comparison of the expression levels of inflammatory cytokines between patients carrying mutations located within EGFr domains 1-6 and those carrying mutations located within EGFr domains 7-34**

|  | EGFr 1-6 | EGFr 7-34 | t/z/χ^2^ | *P* | *P*-adj |
| --- | --- | --- | --- | --- | --- |
| Age | 43.06 ± 10.98 | 40.60 ± 10.20 | -1.869 | 0.067 |  |
| Gender | 16/16 | 5/11 | 0.029 | 0.865 |  |
| INF-γ | 1.83 (0.34-6.98) | 2.07 (0.514-6.13) | -0.937 | 0.349 | 0.524 |
| TNF-α | 3.66 (0.83-11.49) | 4.4 (1.13-10.32) | -0.627 | 0.530 | 0.707 |
| TNF-β | 1.35 (0.72-2.48) | 1.42 (0.75-2.48) | -0.418 | 0.676 | 0.676 |
| IL-1β | 2.38 (0.55-5.43) | 2.9 (0.92-6.23) | -1.118 | 0.263 | 1.052 |
| IL-2 | 2.26 (0.59-5.23) | 2.75 (0.69-5.78) | -1.100 | 0.271 | 0.650 |
| IL-4 | 3.43 (0.60-14.79) | 5.92 (1.16-18.20) | -1.491 | 0.136 | 1.632 |
| IL-5 | 2.35 (0.81-9.11) | 3.16 (0.87-7.42) | -1.109 | 0.267 | 0.801 |
| IL-6 | 5.44 (1.52-12.77) | 5.73 (1.25-16.65) | -0.591 | 0.555 | 0.666 |
| IL-8 | 6.72 (2.01-21.34) | 8.39 (3.18-18.03) | -1.000 | 0.317 | 0.543 |
| IL-10 | 2.21 (0.79-6.60) | 3.03 (1.08-8.7) | -1.018 | 0.309 | 0.618 |
| IL-17F | 1.24 (0.25-4.28) | 1.34 (0.47-3.41) | -1.355 | 0.175 | 1.050 |
| IL-22 | 2.69 (0.87-7.81) | 3.18 (0.89-7.88) | -0.464 | 0.643 | 0.701 |

Abbreviations: INF- γ: interferon-γ, TNF: Tumor necrosis factor, IL: interleukin.

Notes: the expression of inflammatory cytokines was shown as median and range.

Notes: *P*-adj were FDR-adjusted

**Table SIII. Correlation between the expression levels of inflammatory cytokines and mRS in patients with CADASIL**

|  | Prescence of abnormal mRS scores | |
| --- | --- | --- |
|  | OR (95% CI) | *P* |
| IFN-γ | - | 0.912 |
| TNF-α | - | 0.521 |
| TNF-β | 5.074 (1.323-19.461) | 0.018^*^ |
| IL-1β | - | 0.444 |
| IL-4 | - | 0.327 |
| IL-5 | - | 0.537 |
| IL-6 | - | 0.214 |
| IL-8 | - | 0.160 |
| IL-17F | - | 0.691 |
| IL-22 | - | 0.067 |

Abbreviations: CADASIL: cerebral autosomal dominant arteriopathy with subcortical infarcts and leukoencephalopathy, mRS: modified Rankin scale, OR: odds ratio, 95% CI: 95% confidence interval.

Notes: Data were analyzed using univariate binary logistic regression. ^*^Indicates a significant association.

**Table SIV. Correlation between the** **expression levels of** **inflammatory cytokines and cognitive efficiency in patients with CADASIL**

|  | MoCA | | MMSE | |
| --- | --- | --- | --- | --- |
|  | β (95% CI) | *P* | β (95% CI) | *P* |
| IFN-γ | 0.112 (-0.820-1.707) | 0.482 | 0.059 (-0.673-1.029) | 0.421 |
| TNF-α | 0.024 (-0.800-0.932) | 0.878 | -0.011 (-0.588-0.546) | 0.941 |
| TNF-β | -0.305 (-8.897--0.008) | 0.050 | -0.283(-6.118--0.112 | 0.042* |
| IL-1β | -0.058 (-1.948-1.346) | 0.714 | -0.060 (-1.269-0.826) | 0.673 |
| IL-4 | -0.022 (-0.600-0.522) | 0.889 | -0.044(-0.426-0.313) | 0.759 |
| IL-5 | 0.067 (-0.848-1.296) | 0.675 | 0.041 (-0.607-0.813) | 0.772 |
| IL-6 | -0.066 (-0.728-0.479) | 0.679 | -0.054 (-0.467-0.318) | 0.706 |
| IL-8 | 0.054 (-0.392-0.550) | 0.736 | -0.020 (-0.332-0.288) | 0.888 |
| IL-17F | -0.023 (-2.331-2.017) | 0.885 | -0.042 (-1.653-1.228) | 0.769 |
| IL-22 | -0.046 (-1.456-1.088) | 0.772 | -0.077 (-1.017-0.581) | 0.586 |

Abbreviations: CADASIL: cerebral autosomal dominant arteriopathy with subcortical infarcts and leukoencephalopathy, INF- γ: interferon-γ, TNF: Tumor necrosis factor, IL: interleukin, MoCA: Montreal cognitive assessment, MMSE: mini-mental state examination, 95% CI: 95% confidence interval.

Notes: Data were analyzed using univariate linear regression, ^*^Indicates a significant association.

**Table SV. Correlation between the** **expression levels of** **inflammatory cytokines and neuropsychiatric symptoms in patients with CADASIL**

|  | Apathy scores | | SDS scores | |
| --- | --- | --- | --- | --- |
|  | β (95% CI) | *P* | β (95% CI) | *P* |
| IFN-γ | 0.181 (-0.826-3.277) | 0.235 | -0.119 (-2.334-1.026) | 0.437 |
| TNF-α | 0.224 (-0.324-2.255) | 0.138 | 0.077 (-0.801-1.340) | 0.614 |
| TNF-β | 0.388 (2.554-16.328) | 0.008^*^ | 0.171(-2.603-9.344) | 0.261 |
| IL-1β | 0.141 (-1.346-3.669) | 0.356 | 0.064 (-1.626-2.476) | 0.678 |
| IL-4 | 0.213 (-0.256-1.508) | 0.160 | -0.092 (-0.949-0.509) | 0.547 |
| IL-5 | 0.214 (-0.499-2.989) | 0.157 | -0.114 (-1.976-0.902) | 0.456 |
| IL-6 | 0.193 (-0.332-1.519) | 0.203 | -0.132 (-1.086-0.431) | 0.388 |
| IL-8 | 0.094 (-0.542-1.022) | 0.539 | -0.005 (-0.648-0.626) | 0.973 |
| IL-17F | 0.119 (-2.154-4.916) | 0.435 | -0.215 (-4.840-0.801) | 0.156 |
| IL-22 | 0.144 (-1.036-2.892) | 0.346 | -0.097 (-2.111-1.094) | 0.526 |

Abbreviations: CADASIL: cerebral autosomal dominant arteriopathy with subcortical infarcts and leukoencephalopathy, INF- γ: interferon-γ, TNF: Tumor necrosis factor, IL: interleukin, SDS: self-rating depression scale, 95% CI: 95% confidence interval.

Notes: Data were analyzed using univariate linear regression, ^*^Indicates a significant association.

**Table SVI. Correlation between the** **expression levels of inflammatory cytokines and the number of CMBs in patients with CADASIL**

|  | CMBs in deep region | | CMBs in lobar region | |
| --- | --- | --- | --- | --- |
|  | β (95% CI) | *P* | β (95% CI) | *P* |
| IFN-γ | 0.119 (-2.963-7.281) | 0.401 | 0.210 (-1.587-11.375) | 0.136 |
| TNF-α | 0.26 (-0.610-6.109) | 0.107 | 0.296 (0.393-8.858) | 0.033 |
| TNF-β | 0.314 (2.989-39.461) | 0.023^*^ | 0.433 (15.363-59.857) | 0.001^*^ |
| IL-1β | 0.113 (-3.789-8.863) | 0.424 | 0.222 (-1.581-14.371) | 0.114 |
| IL-4 | 0.254 (-164-4.139) | 0.069 | 0.250 (-0.255-5.280) | 0.074 |
| IL-5 | 0.204 (-1.116-7.264) | 0.147 | 0.186 (-1.811-8.998) | 0.188 |
| IL-6 | 0.295 (0.200-4.712) | 0.033* | 0.272 (-0.009-5.830) | 0.051 |
| IL-8 | 0.196 (-0.549-3.135) | 0.165 | 0.178 (-0.858-3.891) | 0.206 |
| IL-17F | 0.163 (-3.587-13.485) | 0.259 | 0.233 (-1.681-19.927) | 0.096 |
| IL-22 | 0.227 (-0.850-8.607) | 0.106 | 0.259 (-0.341-11.710) | 0.064 |

Abbreviations: CADASIL: cerebral autosomal dominant arteriopathy with subcortical infarcts and leukoencephalopathy, INF- γ: interferon-γ, TNF: Tumor necrosis factor, IL: interleukin, CMBs: cerebral microbleeds, 95% CI: 95% confidence interval。

Notes: Data were analyzed using univariate linear regression, ^*^Indicates a significant association.

**Table SVII. Correlation between the** **expression levels of inflammatory cytokines and the ARWMC scores in patients with CADASIL**

|  | ARWMC scores of white matter | | ARWMC scores of basal ganglia | |
| --- | --- | --- | --- | --- |
|  | β (95% CI) | *P* | β (95% CI) | *P* |
| IFN-γ | -0.015 (-0.805-0.722) | 0.913 | -0.073 (-0.338-0.199) | 0.606 |
| TNF-α | 0.027 (-0.461-0.560) | 0.847 | -0.073 (-0.226-0.133) | 0.605 |
| TNF-β | 0.151 (-1.300-4.322) | 0.285 | 0.184 (-0.335-1.636) | 0.191 |
| IL-1β | 0.054 (-0.762-1.120) | 0.704 | -0.038 (-0.377-0.287) | 0.787 |
| IL-4 | 0.095 (-0.217-0.438) | 0.502 | 0.034 (-0.102-0.130) | 0.812 |
| IL-5 | 0.044 (-0.535-0.731) | 0.758 | 0.014 (-0.213-0.234) | 0.923 |
| IL-6 | 0.084 (-0.245-0.451) | 0.555 | -0.046 (-0.143-0.103) | 0.746 |
| IL-8 | 0.103 (-0.176-0.378) | 0.467 | -0.038 (-0.111-0.085) | 0.788 |
| IL-17F | 0.055 (-1.029-1.528) | 0.697 | 0.022 (-0.417-0.486) | 0.879 |
| IL-22 | 0.156 (-0.314-1.106) | 0.268 | 0.129 (-0.136-0.366) | 0.362 |

Abbreviations: CADASIL: cerebral autosomal dominant arteriopathy with subcortical infarcts and leukoencephalopathy, INF- γ: interferon-γ, TNF: Tumor necrosis factor, IL: interleukin, ARWMC: age-related white matter changes, 95% CI: 95% confidence interval.

Notes: Data were analyzed using univariate linear regression,

**Table SVIII. Correlation between the** **expression levels of** **inflammatory cytokines and the number of lacunes in patients with CADASIL**

|  | Lacunes in white matter | | Lacunes in basal ganglia | |
| --- | --- | --- | --- | --- |
|  | β (95% CI) | *P* | β (95% CI) | *P* |
| IFN-γ | -0.063 (-2.015-1.280) | 0.656 | -0.161 (-0.689-0.187) | 0.254 |
| TNF-α | -0.016 (-1.166-1.042) | 0.911 | -0.042 (-0.340-0.253) | 0.769 |
| TNF-β | 0.150 (-2.824-9.331) | 0.287 | 0.143 (-0.806-2.464) | 0.313 |
| IL-1β | 0.065 (-1.564-2.503) | 0.645 | 0.092 (-0.367-0.723) | 0.515 |
| IL-4 | 0.214 (-0.160-1.231) | 0.129 | 0.136 (-0.098-0.281) | 0.336 |
| IL-5 | 0.077 (-0.994-1.738) | 0.587 | -0.002 (-0.371-0.365) | 0.986 |
| IL-6 | 0.157 (-0.328-1.165) | 0.265 | 0.143 (-0.099-0.303) | 0.311 |
| IL-8 | 0.050 (-0.495-0.706) | 0.726 | -0.045 (-0.187-0.136) | 0.752 |
| IL-17F | 0.074 (-2.034-3.486) | 0.600 | -0.038 (-0.842-0.645) | 0.791 |
| IL-22 | 0.207 (-0.390-2.651) | 0.142 | 0.181 (-0.145-0.677) | 0.200 |

Abbreviations: CADASIL: cerebral autosomal dominant arteriopathy with subcortical infarcts and leukoencephalopathy, INF- γ: interferon-γ, TNF: Tumor necrosis factor, IL: interleukin, 95% CI: 95% confidence interval.

Notes: Data were analyzed using univariate linear regression,
